# Supplementary material for: Understanding Enablers and Barriers to the Implementation of Nutrition Standards in Publicly Funded Institutions in Victoria
Source: Nutrients. 2022 Jun 24;14(13):2628. doi: 10.3390/nu14132628 (PMC9268167; doi:10.3390/nu14132628)
Supplement: Supplementary file 1 [file nutrients-14-02628-s001.zip › nutrients-1769809-supplementary.pdf]

Supplementary Table S1. Participant responses to the pre-interview questionnaire by employing publicly-funded institution

Questionnaire	Health (n=15)	School (n=10)	Workplace (including sport and recreation) (n=11)	Manufacturer/ supplier/ retailer (n=5)	Total (n=41)
**Organisational characteristics**					
**Number of staff/students**					
**<1000**	6	9	0	2	21
**1000-9999**	6	1	0	1	13
**≥10000**	1	1	3	0	5
**Health's level of priority**					
**High priority**	10	5	5	5	25
**Moderate priority**	4	3	4	0	10
**Low priority**	1	2	1	0	4
**Not a priority**	0	0	0	0	0
**Healthy eating's level of priority**					
**High priority**	3	4	2	5	14
**Moderate priority**	9	2	5	0	16
**Low priority**	2	3	3	0	8
**Not a priority**	1	1	0	0	2
**General**					
**Aware of Guidelines/Policy**	15	10	11	5	41
**Knows where to access Guidelines/Policy**	15	9	11	5	40
**Stage of implementation of Guidelines/Policy**					
**Fully implemented**	2	4	2	1	9
**In the process of implementing**	7	4	8	1	19
**Planning to implement**	3	1	0	1	5
**Aware of Healthy Eating Advisory Service**	15	10	11	5	41
**Received support from HEAS**	14	6	11	4	35
**Aware of Cancer Council Victoria Achievement Program (AP)**	11	7	10	1	29
**Received support from Cancer Council Victoria AP**	5	6	5	0	15
**Aware of other policies/guidelines**	6	4	6	3	19
**Implementation**					
**Organisation sells/provides food**	15	10	11	4	40
**Methods of food provision**					
Catering	1	0	0	0	1
Catering, Fundraising	1	1	1	0	3
Food outlet	0	1	0	0	1
Food outlet, Catering	0	4	3	0	7
Food outlet, Catering, Fundraising	1	0	0	0	1
Food outlet, Fundraising	0	3	0	0	3
Food outlet, Vending machine, Catering	4	0	2	1	7
Food outlet, Vending machine, Catering, Fundraising	7	0	2	1	10
Fundraising, Other	0	1	0	0	1
Other	0	0	1	2	3
Vending machine	1	0	0	0	1
Vending machine, Catering	0	0	1	0	1
Vending machine, Catering, Fundraising	0	0	1	0	1
**Nutrition composition of foods assessed**	15	8	11	4	37
**Changes made to improve nutritional composition of foods**	15	8	11	5	39

**Identified barriers to implementing Guidelines/Policy**	15	8	11	4	38
**Identified enablers to implementing Guidelines/Policy**	13	4	9	4	30
**Monitoring changes and evaluating impact**	13	8	11	2	34
**Process Outcomes**					
**Adequate support by organisation**	9	7	7	4	27
**Adequate resources provided by organisation**	6	7	6	3	22
